# Edible Origami Actuators Using Gelatin-Based Bioplastics

**DOI:** 10.1021/acsapm.3c00919

**Published:** 2023-07-06

**Authors:** Spencer J. Matonis, Bozhong Zhuang, Ailla F. Bishop, Durva A. Naik, Zeynep Temel, Christopher J. Bettinger

**Affiliations:** Carnegie Mellon University, 5000 Forbes Ave., Pittsburgh, Pennsylvania 15213, United States

**Keywords:** edible, origami, gelatin, bioplastic, actuator, moisture

## Abstract

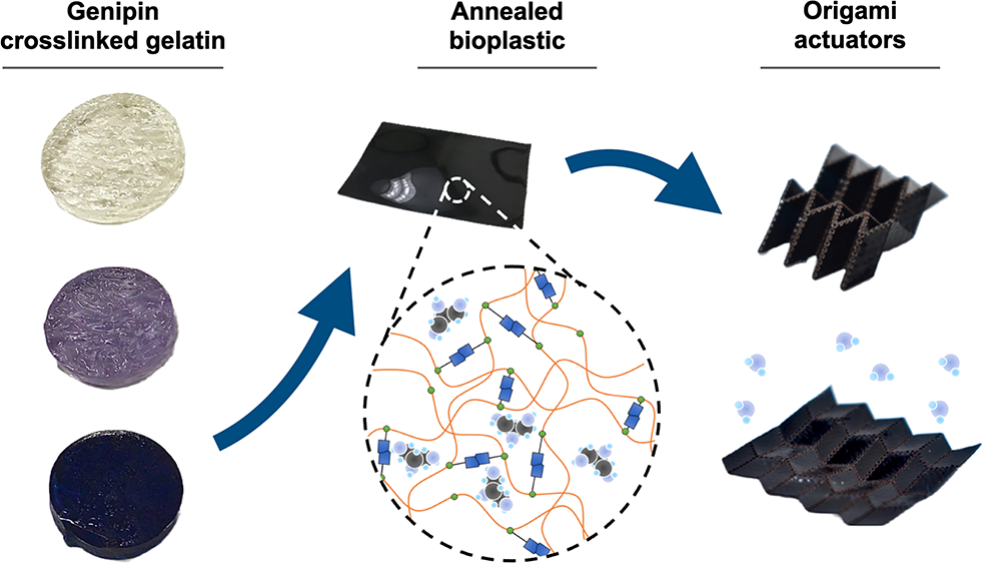

The potential of ingestible medical devices can be greatly enhanced through the use of smart structures made from stimuli-responsive materials. While hydration is a convenient stimulus for inducing shape changes in biomaterials, finding robust materials that can achieve rapid actuation, facile manufacturability, and biocompatibility suitable for ingestible medical devices poses practical challenges. Hydration is a convenient stimulus to induce shape changes in smart biomaterials; however, there are many practical challenges to identifying materials that can achieve rapid actuation and facile manufacturability while satisfying constraints associated with biocompatibility requirements and mechanical properties that are suitable for ingestible medical devices. Herein, we illustrate the formulation and processability of a moisture-responsive genipin-crosslinked gelatin bioplastic system, which can be processed into complex three-dimensional shapes. Mechanical characterization of bioplastic samples showed Young’s Modulus values as high as 1845 MPa and toughness values up to 52 MJ/m^3^, using only food-safe ingredients. Custom molds and UV-laser processing enabled the fabrication of centimeter-scale structures with over 150 independent actuating joints. These self-actuating structures soften and unfold in response to surrounding moisture, eliminating the need for additional stimuli or actuating elements.

## Introduction

One only has to look out their window to draw inspiration from a beautifully integrated portfolio of biological actuators. In nature, we find elegant structures, capable of rapid reconfiguration in response to external stimuli, composed of only a handful materials. Some of the best examples of these natural smart materials include the fast-actuating skin of cuttlefish,^1^ swelling-based actuation of plant stomata,^2^ or the shape memory response of tendon fibrils.^3−5^ In contrast, human-made stimulus-responsive structures are often limited by tedious and expensive manufacturing techniques, toxic constituents, and slow response times relative to their natural counterparts.^6,7^

Hydration is a simple, yet effective actuation stimulus, which can be aptly leveraged by permeable, hydrophilic materials. Biopolymer-based hydrogel actuators are particularly advantageous in applications where ultracompliant and biocompatible materials are required or external power sources are impractical, such as internal medicine. As a result, hydrogels with swelling ratios of up to 1000× applied to a great effect for tissue engineering, drug delivery, and wound repair.^8−13^ Some examples of biopolymer-based hydrogels include cellulose, chitosan, polysaccharides (agarose, alginate, and starch), and proteins like collagen or gelatin.^14^ Despite their accessibility and biocompatibility, these materials may struggle to achieve wide-scale adoption as moisture-responsive actuators due to slow response kinetics, hysteresis, chemical degradation, and insufficient Young’s moduli.^15−17^

Crosslinking agents are an excellent tool for tuning the properties of native hydrogel systems for structural applications. Crosslinking agents act to bond a hydrogel’s three-dimensional network of polymer chains with both physical (e.g., chain entanglement, hydrogen bonding, etc.) and chemical interactions (covalent bonding).^18,19^ Chemically crosslinked hydrogels exhibit increased Young’s moduli and slower *in vivo* degradation kinetics.^20,21^ However, many crosslinkers such as aziridine or glutaraldehyde are cytotoxic.^22,23^

Genipin is a naturally derived nontoxic crosslinker that reacts with free amines including those found in lysine, arginine, and glutamine. Extracted from*Gardenia jasminoides*, genipin is approved for consumption by the U.S. Food and Drug Administration (FDA) as a food colorant, and genipin is both biocompatible and 10,000 times less cytotoxic than glutaraldehyde.^24,25^ Genipin has been used extensively as a crosslinker for biopolymers such as gelatin. Genipin-crosslinked gelatin, termed gelapin, has been used as a tissue engineering scaffold and a drug delivery matrix.^26−28^ While the chemical, physical, and optical properties of gelapin films have also been characterized, the parameter space for the mechanical properties of gelapin-based structures at the device scale has been under-reported.^29,30^

Here, we describe a plasticized gelapin bioplastic, which is amenable to many material processing techniques to create hydration-responsive actuators. Programmable hydrophilic substrates of this nature have previously been reported with basic locomotion and gripping functions with a limited number of linkages.^31−33^ The introduction of origami-inspired design to smart substrates has enabled far more complicated mechanisms, giving a greater range of motion to actuating structures.^34−36^

In this work, the robust mechanical properties afforded to gelapin-based bioplastics permit manufacturing of three-dimensional moisture-sensitive structures by sheet molding and laser etching. To demonstrate this versatility, we fabricated a range of origami actuator designs that rapidly unfold in response to elevated ambient humidity. Dynamic mechanical properties enable the deployed substrate to delicately conform to irregular or dynamic surfaces. As demonstrated by Kuribayashi et al., these features make self-deployable origami systems particularly well-suited for internal medical applications, where they may omit the need for more invasive tools such as endoscopes or catheters.^37^ Only recently has this concept been translated to the gastrointestinal track, where origami structures may be orally ingested to facilitate internal medicine.^38^

## Experimental Section

### Materials

Glycerol, phosphate-buffered saline (PBS) solution, and type A, 300-Bloom factor porcine gelatin were obtained from Millipore-Sigma (Milwaukee, WI, USA) and used as received unless otherwise noted. Gold shellac flakes were purchased from Waymil LLC (Doral, FL) and used as received. Purified genipin powder (Wilshire; Princeton, NJ, USA) was mechanically ground and sifted to produce microparticles with the largest dimension of <53 μm.

### Bioplastic Film Preparation

Gelatin was first hydrated in a 15% gelatin, 85% DI water (by wt.) solution for 30 min. Glycerol was incorporated into this bloom mixture, most commonly at 37.5 wt % (gelatin basis). The mixture was covered and heated in a 60 °C oven for 20 min to create a homogeneous melt. To reach the desired crosslinking percentage, genipin powder was dissolved in a solution composed of 70% ethanol and 30 wt % DI water equal to half of the bloom mixture mass. The genipin solution was slowly incorporated into the gelatin melt, which was maintained at 45 °C on a hot plate and stirred at roughly 300 rpm. This mixing process lasted for 3–5 min while the crosslinking reaction was initiated and the glycerol plasticizer was uniformly dispersed. The final solution was poured into a glass sheet mold coated with Teflon tape and annealed for 24 h at approximately 35 °C to accelerate genipin crosslinking and evaporate the majority of the material’s water and ethanol contents (>95 wt %). To mold biogel samples, all preparation remained the same, while a final curing stage was performed at room temperature to retain the water content (in place of annealing). The genipin crosslinking concentration (mol %) was calculated on a per amine group basis as measured through ninhydrin assays (Figure S1). To prevent undesired hydration or degradation, long-term sample storage was done in an air-sealed environment with desiccant material.

### Mechanical Testing

Uniaxial tensile tests were conducted using a single-column Instron universal testing system (100 N static load cell, Instron model no. 5943; Norwood, MA) with samples cut to ASTM type-V dog bone standards using a rabbit CO_2_ laser (Middletown, Ohio). Sample batches were tested in triplicate and strained at a rate of 10 mm/min until fracture. Reported Young’s modulus values were calculated as the slope of stress–strain curves at a tensile strain of 0.002.

### Swelling and Degradation Test

In order to test the swelling and degradation rates of gelapin biogel samples at varying crosslinking percentages, 50 mg samples were cut and sealed in vials containing 10 mL of 0.01 M PBS solution and immersed in a 37 °C water bath. Samples were extracted at regular intervals for weighing, with excess water carefully removed from the sample surface. All formulation batches were tested in triplicate.

### Thermal Characterization

Differential scanning calorimetry (DSC) was performed with a TA Instruments Q20 DSC (New Castle, DE). Plastic samples were dehydrated in vacuum for 24 h prior to testing. Upon loading and sealing into aluminum sample pans, 5 mg samples were cooled to 0 °C, heated to 200 °C, and cooled back down to 0 °C at a rate of 10 °C/min. Three heat flow cycles were conducted per sample run with nitrogen gas flowing through the testing chamber at a rate of 50 mL/min.

### Humidity Response Characterization

Gelapin material, bioplastic strips (50 × 10 × 0.15 mm) were prepared via CO_2_ laser cutting. Samples were folded in half and fixed onto a platform on one side (link 1) before being compressed with a 5 kg weight inside a humidity chamber at 26 °C and a constant relative humidity (RH) of 30%. Humidity levels were increased in 10% RH increments across multiple sample runs up to 80% RH. After 30 min, the weight was removed to release the folded bioplastic.

## Results and Discussion

### Formulation Strategy and Film Mechanical Properties

**Figure 1:**
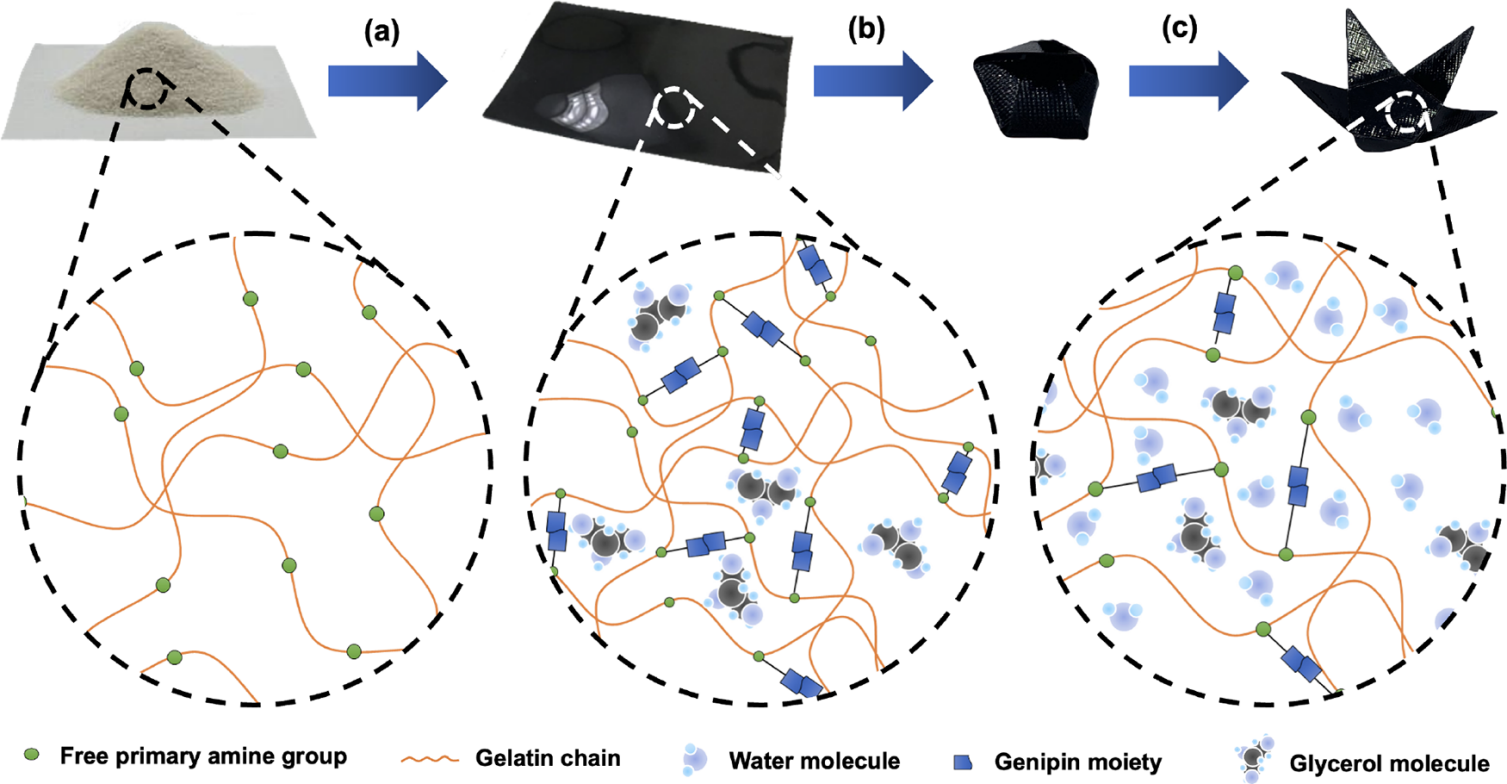
Schematic of the edible origami production process and associated molecular interactions. (a) Synthesis of the bioplastic substrate with genipin crosslinking, glycerol plasticization, and thermal annealing. (b) UV-laser etching and compression folding of compact origami structures. (c) Hydration and deployment of origami structures in ambient humidity.

**Figure 2:**
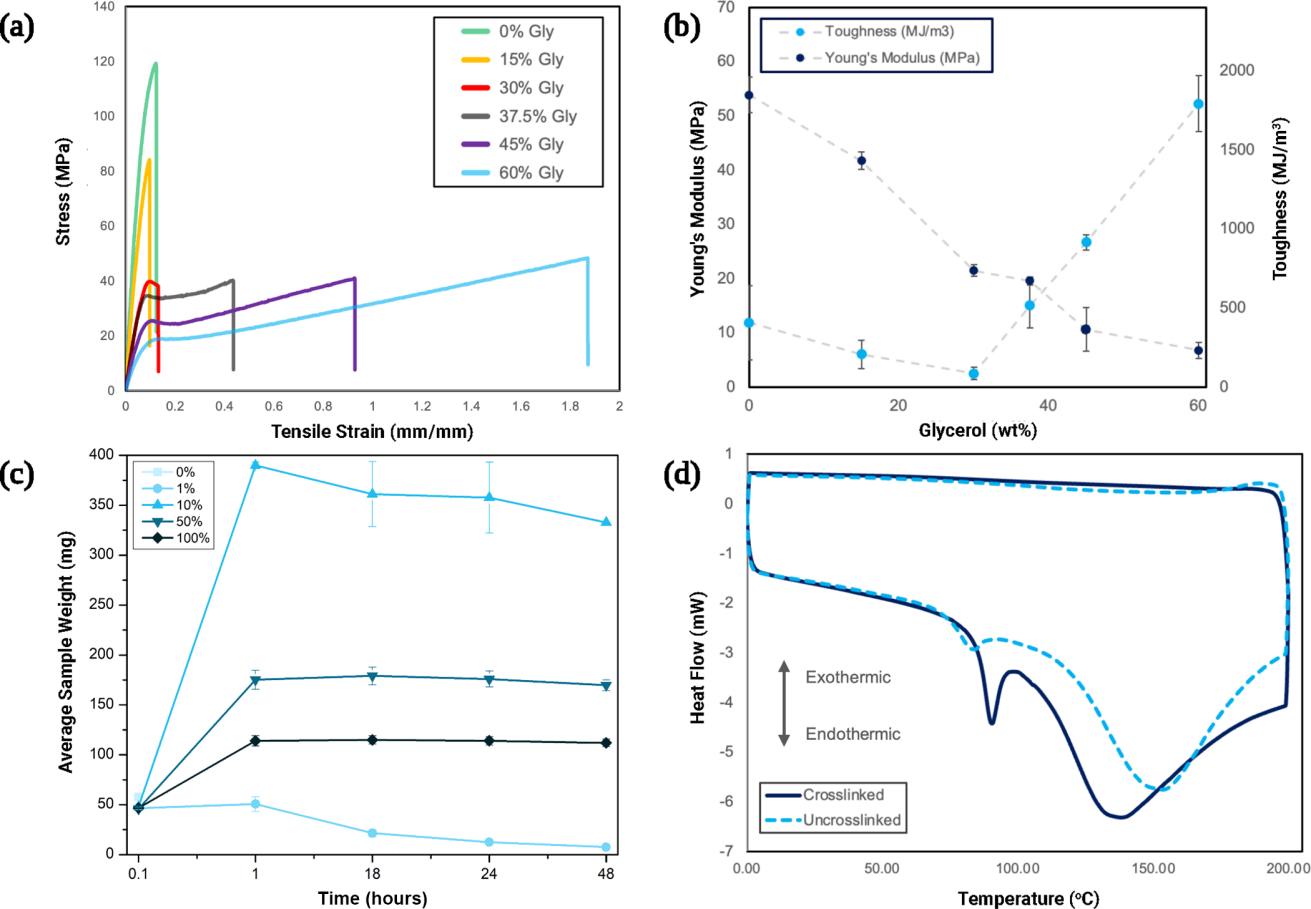
Stress–strain plot (a) and summary (b) of uniaxial tensile testing results for samples with varying glycerol concentration by weight relative to gelatin. (c) Swelling and degradation in 37 °C PBS solution over 48 h vs genipin crosslinking concentration. (d) DSC thermogram showing 100 mol % crosslinked and 0 mol % crosslinked sample heat flow (mW).

Gelatin is produced through the thermal denaturation and partial hydrolysis of animal-based collagen. To create the gelatin’s sol–gel state (below 37 °C), residual collagen helices facilitate a network of physically crosslinked intermolecular junctions.^39^ Once a gelatin gel is dehydrated, these junctions impede slippage between polymer chains and result in a brittle film with low toughness and extensibility.^40^ Plasticizers increase intermolecular spacing, reduce intramolecular hydrogen bonds, and thus counter this effect.^41,42^ Here, we have selected glycerol as an abundant and edible plasticizer to increase the ductility and toughness of gelapin bioplastic (Figure 1). As shown in Figure 2a,b, the uniaxial tensile testing results demonstrate a strong correlation between the glycerol concentration (by wt. relative to gelatin) and film mechanical properties. Specifically, increasing the glycerol content from 0 to 60 wt % increases the toughness from 11.82 ± 6.9 to 52.2 ± 5.18 MJ/m^3^ and decreases the Young’s modulus from 1845 ± 113 to 232 ± 49.7 MPa. The ideal material for self-deployable edible origami structures would accommodate deformation along the designated folding pattern without brittle fracture and maximize stored strain energy to facilitate actuation. To achieve this behavior, a glycerol concentration of 37.5 wt % was selected with a combination of elasticity (671 ± 26 MPa) and toughness (15 ± 4.1 MJ/m^3^).

Consistent with other crosslinking reagents, an increased genipin loading was found to slow the degradation and swelling kinetics of unannealed gelapin (Figure 2c).^43−45^ The percent crosslinked figure approximates the bonding saturation of free amine groups in the gelatin biopolymer network, with two genipin molecules consumed per bonding of every site pair. While relatively uncrosslinked samples with a genipin concentration of ≤1 mol % degraded in under 48 h, 10 mol % crosslinked samples had enough network stability to allow for significant swelling (up to 713%) after 1 h. This effect is diminished as crosslinking percentage increases, with fully crosslinked samples swelling by 150% over 48 h, likely due to a smaller mesh size preventing the infiltration of surrounding fluid.^46,47^ Unlike their biogel counterparts, 100 mol % crosslinked bioplastic samples showed no detectable degradation after 90 days in PBS (Figure S2). Gelatin bioplastics degrade and disintegrate within 24 days in a simulated gastric environment (pH = 1.2). Due to the greater strength and environmental stability of crosslinked samples, we settled on using a 100 mol % genipin crosslinking saturation for further samples.

Thermal annealing accelerates genipin crosslinking kinetics and reduces the water content in the network, two processes that are critical to the mechanical properties of gelapin bioplastics. Differential scanning calorimetry (DSC) thermograms on dehydrated samples show a denaturation temperature of 92 °C for crosslinked material, while native gelatin films show a less pronounced denaturation signature at approximately 84 °C. These results corroborate previous findings showing that genipin and glutaraldehyde crosslinking increases the thermostability of gelatin systems.^48,49^ Conversely, the gelapin bioplastic shows a higher denaturation enthalpy compared to pristine gelatin. Subsequent FTIR absorption analysis (Figure S3) shows a significant reduction in amide A peaks associated with O–H stretch in unplasticized, crosslinked samples. Additionally, the presence of glycerol suggests significant enhancement in the amide III region (1030 cm^–1^ peak). This region tends to reflect C–N stretching vibrations coupled to the N–H bend and may correlate to an increase in triple helix formation.^50,51^

### Humidity Response of Bioplastic Actuators

**Figure 3:**
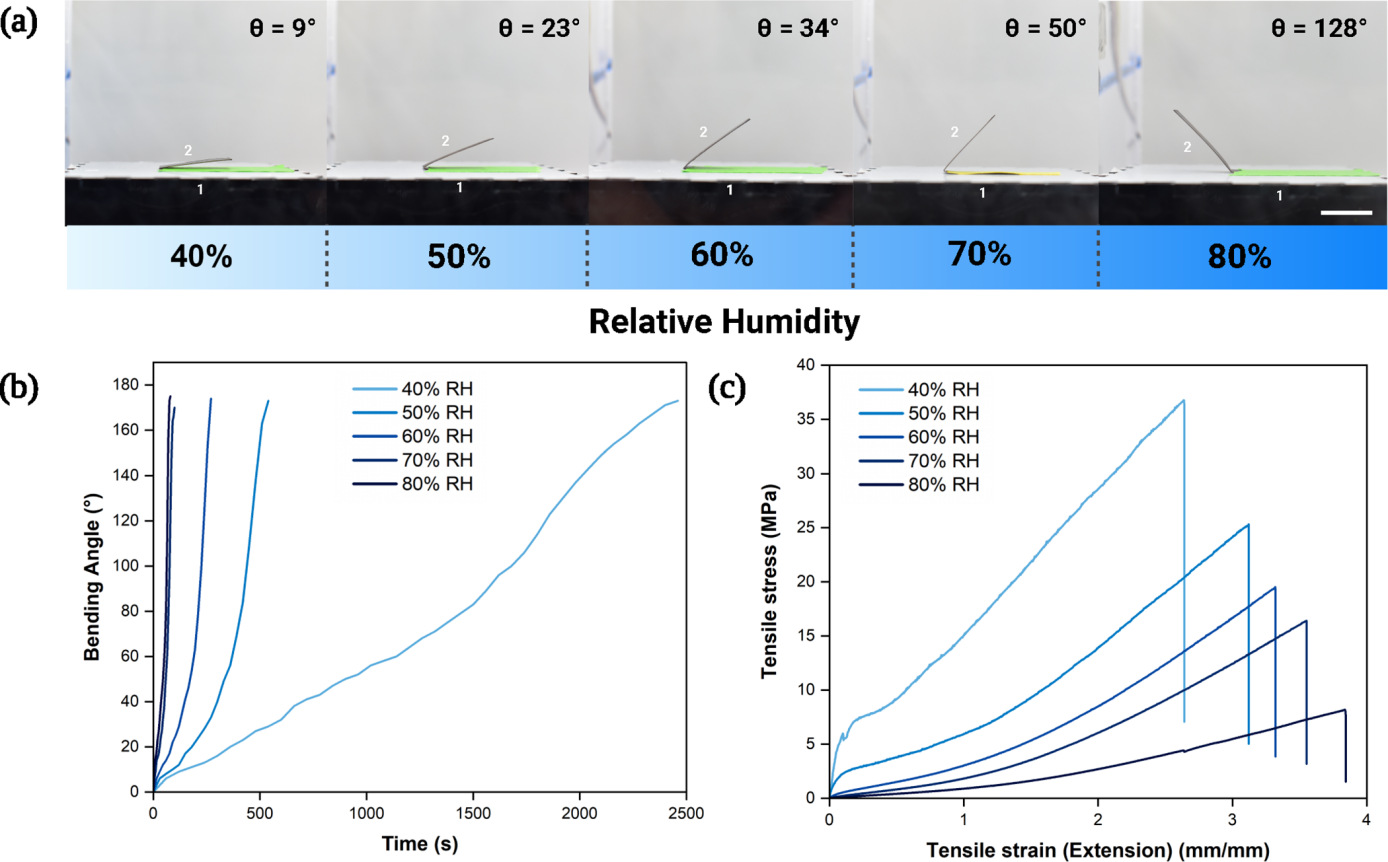
(a) Profile images of bioplastic samples at varying levels of ambient humidity (2 cm scale bar). Each photo was taken 90 s after the samples were released. In the two-linkage system, link one is notated as the fixed end, and link 2 is notated as the free end. (b) Bending angle plotted with respect to time for beam samples at varying humidity levels. (c) Stress–strain plots of bioplastic samples having been equilibrated at varying levels of ambient humidity for 1 h.

Unfolding speeds were measured using crosslinked gelapin formulations (100 mol % crosslinking, 37.5 wt % glycerol) as a function of ambient humidity (Figure 3a). The folding angle (measured between links 1 and 2) was tracked and plotted (Figure 3b) to calculate the time to achieve a 160° actuation angle as a function of relative humidity (2248 and 69 s for 40 and 80% RH, respectively). Next, samples underwent tensile testing after being equilibriated for 1 h at a range of 40–80% RH. Bioplastic samples at 40% RH exhibited a yield stress of 5.67 ± 0.42 MPa, which is comparable to some starch-based bioplastics^52^ (Figure 3c) to a hyperelastic model at 50% RH and higher (more commonly seen in elastomeric substrates^53,54^). Stress–relaxation experiments were performed at moderate humidity levels (30–80% RH) and reflect a similar trend in relaxation behavior relative to ambient moisture (Figure S4). One possible explanation for this trend is that the hydrophilic amino and carboxyl groups in gelatin polymers induce intermolecular hydration, which can also occur with polyol groups in glycerol.^55^ Gelapin hydration increases chain mobility thus allowing the network to return to its prestrained state.

Hydration-based actuation is convenient, but premature deployment due to environmental moisture is a concern that restricts storage environments and constrains *in vivo* deployment in certain contexts. In applications where delayed humidity actuation is required, hydrophobic coatings and packaging can be used to obstruct the infiltration of water molecules from the atmosphere.^56^ Multiple experiments were conducted using a shellac-based hydrophobic coating to control the short-term moisture sensitivity of the base material (Figure S5). Shellac was specifically selected for its biocompatibility and adhesive properties as a material with low water permeability.^57−59^ Shellac-coated gelapin bioplastics submerged in liquid water for up to 1 min retained comparable values of the original yield stress (15–20 MPa) and failure stress (30–40 MPa). Comparatively, uncoated samples were found to revert to a softened state with no yield stress and a roughly 400% reduction in failure stress. In future work, we may leverage a similar style of hydrophobic drug-based coatings to create origami-based resident drug-delivery systems for internal medicine.^60,61^

**Figure 4:**
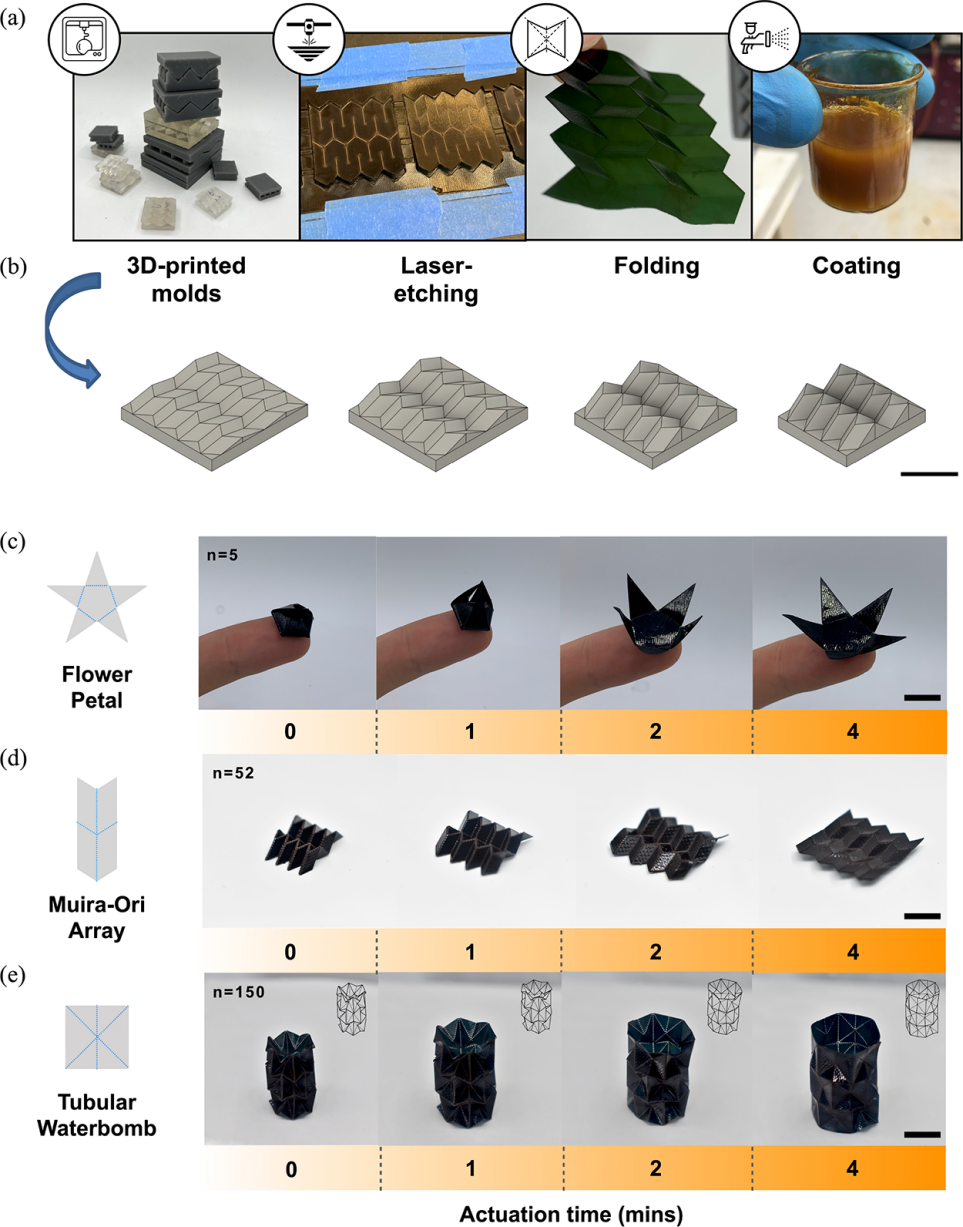
(a,b) Gelapin bioplastics are amenable to various postprocessing techniques. Compression molds (bottom piece) for the Miura origami array arranged in a sequence of low to high folding degrees (1 cm scale bar). (c–e) Examples of bio-origami actuators and their deployment speed under 80% RH. “*n*” refers to the number of creases for the whole (1 cm scale bar).

Annealed gelapin bioplastics are easy to handle and amenable to ubiquitous processing techniques such as molding and etching (Figure 4a). Gelapin sheet material can be processed into moisture-responsive sheets thus creating actuating elements. Origami-inspired shapes permit compression into small volumes and subsequent expansion in response to environmental moisture. Custom molds (Figure 4b) combined with UV-laser cutting can create gelapin bioplastics with over 100 creases per 3 cm^2^. Compared to alternative works with individual cm-scale folds, this improved feature density can enable smaller actuator designs with higher degrees of freedom and articulation.^62−64^Figure 4c–e shows the example of bioplastic origami products and their deployment under 80% RH. A 150 μm-thick gelapin substrate containing 37.5 wt % glycerol and 100 mol % genipin crosslinking was used to create the origami. Structures with relatively large feature sizes (>5 mm) were able to be folded manually, by hand (Figure 4c). Structures with smaller feature sizes, such as those depicted in Figure 4d,e, made use of custom resin molds printed using stereolithography (SLA). Laser-cut samples were compressed via clamps for 30 s in a progressive series of molds in order to generate controlled, detailed auxetic folding patterns (Figure 4b). In all designs, buckling was facilitated by a network of 300 μm holes with 400 μm between each hole (Figure S6). Compression molding was conducted at ambient temperatures and an ambient humidity level below 40% RH. Using this technique, features as small as 50 μm should be patternable based on the printing resolution of the compression molds, the UV laser beam width, and the user’s ability to align features. The structural transformations represent irreversible actuation as folded joints in the origami experience a shape memory effect, triggered by a permeation of water molecules into the material. The hydrated bioplastic shows a hyperelastic deformation profile (Figure S5) and releases stored strain energy at the folded joints in an attempt to return to the originally casted, planar configuration. The actuation speed of gelapin-based origami (1–5 min) was found to be comparable to other temperature and hydration-driven self-deployable origami models.^36,37,65,66^ Future work may investigate the lamination of multiple biopolymer layers with opposing shape memory effects to create reversible actuator systems, as researchers have shown in parallel material systems.^67−69^ The actuation speed and force may similarly be improved through the use of prestretched substrates and elastic joints, as part of a composite origami architecture.^70,71^

## Conclusions

Genipin-crosslinked gelatin (“gelapin”) bioplastic films were prepared by thermally accelerated crosslinking and annealing to expand the parameter space of elasticity and toughness in moisture-responsive biopolymer actuators.^72−74^ Characterization of this humidity-responsive actuation was scaled from a single-fold cantilever model to origami-inspired structures of increasing complexity. The accessible processing techniques described in this study and robust mechanical properties of the gelapin bioplastic will enable detailed ingestible device design with high degrees of freedom and tunability. In particular, biodegradable actuators offer unprecedented substrate functionality for deployable and reconfigurable device systems in fields such as internal medicine and soft robotics. With improved hydration resistance, gelapin origami may come to facilitate long-term resident devices in the GI tract. Future work will seek to improve the actuation speed of gelapin origami as well as demonstrate internal drug delivery and bioelectronic applications, which leverage biocompatible and transformable architectures for long-term residence in the gut.

## Supporting Information Available

Ninhydrin assay standard curve, photos of bioplastic degradation, FTIR absorption plots, stress–relaxation data, stress–strain plots of bioplastic samples with and without shellac coatings, and design details and images of origami patterning (PDF)

## Notes

^‡^ S.J.M. and B.Z. contributed equally.
