# Supplementary material for: Edible Origami Actuators Using Gelatin-Based Bioplastics
Source: ACS Appl Polym Mater. 2023 Jul 6;5(8):6288–95. doi: 10.1021/acsapm.3c00919 (PMC10425958; doi:10.1021/acsapm.3c00919)
Supplement: Supplementary file 1 — ap3c00919_si_001.pdf [file ap3c00919_si_001.pdf]

Supporting Information

for

Edible origami actuators using gelatin-based bioplastics

*Spencer J. Matonis, Bozhong Zhang, Ailla F. Bishop, Durva A. Naik, Zeynep Temel, Christopher J. Bettinger**

Carnegie Mellon University, 5000 Forbes Ave, Pittsburgh, PA 15213, USA

*Corresponding author (cbetting@andrew.cmu.edu)

Detailed Methods

Ninhydrin Assay and Standard Curve Formulation. The concentration of free primary amines within 300 bloom factor type A gelatin was determined using ninhydrin assay (**Figure S1**). Briefly, 0.1 gram of gelatin was bloomed in a mixture of 2 ml 0.05% glacial acetic acid and 1 ml deionized water (DI water). Bloomed gelatin mixture was further incubated in 1 ml of ninhydrin reagent (Sigma Aldrich Product No. N7285) at 100 °C for 10 min and then cooled down to 25°C over 45 min. The transparent gelatin mixture turns blue ($\lambda_{Abs\text{-}max}$ = 560 nm) upon reaction of ninhydrin reagent with free primary amines. 1 ml of the sample was further diluted with deionized water (1:19) prior to the absorption measurement. Absorption was measured using UV-vis spectroscopy (UV- 2600, Shimadzu, Kyoto, Japan) and calibrated to tyramine standards (SI figure). Estimated concentration of free primary amines in 1 gram of 300 bloom factor type A gelatin is 0.17 mM.

***In Vitro* Degradation Experiments**. Macroscopic degradation of bioplastics was monitored by placing coupons (approximately 100 mg and 150 um thick) in either PBS or simulated gastric fluid (SGF; pH = 1.2; Ricca Chemical Company, Arlington TX) (**Figure S2**). Degradation samples were stored at 37 °C in 20 ml of SGF in a sterilized vial and monitored daily. Samples were agitated at 60 rpm using an orbital shaker.

FT-IR Spectroscopy. **Spectra were recorded in gelapin samples using** attenuated total reflection Fourier Transform Infrared Spectroscopy (ATR-FTIR). Samples were prepared as powders from lyophilized granules. FTIR spectra were collected four combinations of plasticizer and genipin crosslinker concentration to isolate potential interactions in the biopolymer network. The primary peaks of interest are labeled along with their corresponding bond vibrations (**Figure S3**). All spectra were acquired in the range of 600-4000 cm^{-1} and, for each sample, 32 scans were performed at a resolution of 1 cm^{-1}.

Mechanical Characterization of Gelapin Bioplastics. Stress relaxation plots were collected using an Instron Universal Testing System (Norwood, MA) with samples cut to ASTM Type-V dog bone standards using a Rabbit CO_2 laser (Middletown, OH) (**Figure S4**). Sample batches were tested in triplicate and uniaxially strained to 0.03 at a strain rate of 1 mm/min before being held in tension for 60 s. Samples were normalized at their respective moisture levels for 1 h prior to testing using a Memmert HCP 105 humidity chamber (Eagle, WI). Gelapin bioplastics coated with shellac were prepared as follows. An aqueous solution of shellac was synthesized by dissolving 30 wt% shellac flakes into pure ethanol and stirring in a covered beaker for 18 h. Once gelapin samples were spray coated and dabbed to remove excess solution, they were allowed to dry at room temperature at 20% RH for one hour. Select samples were then immersed in RODI water for one minute prior to uniaxial tensile testing (**Figure S5**).

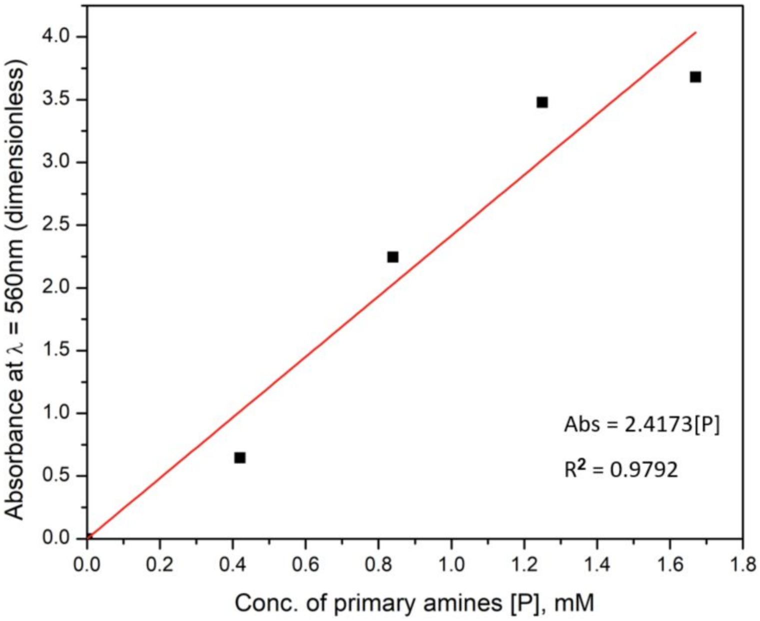

Figure S1: Standard curve from ninhydrin assay to estimate the concentration of primary amines in gelatin/gelapin samples.

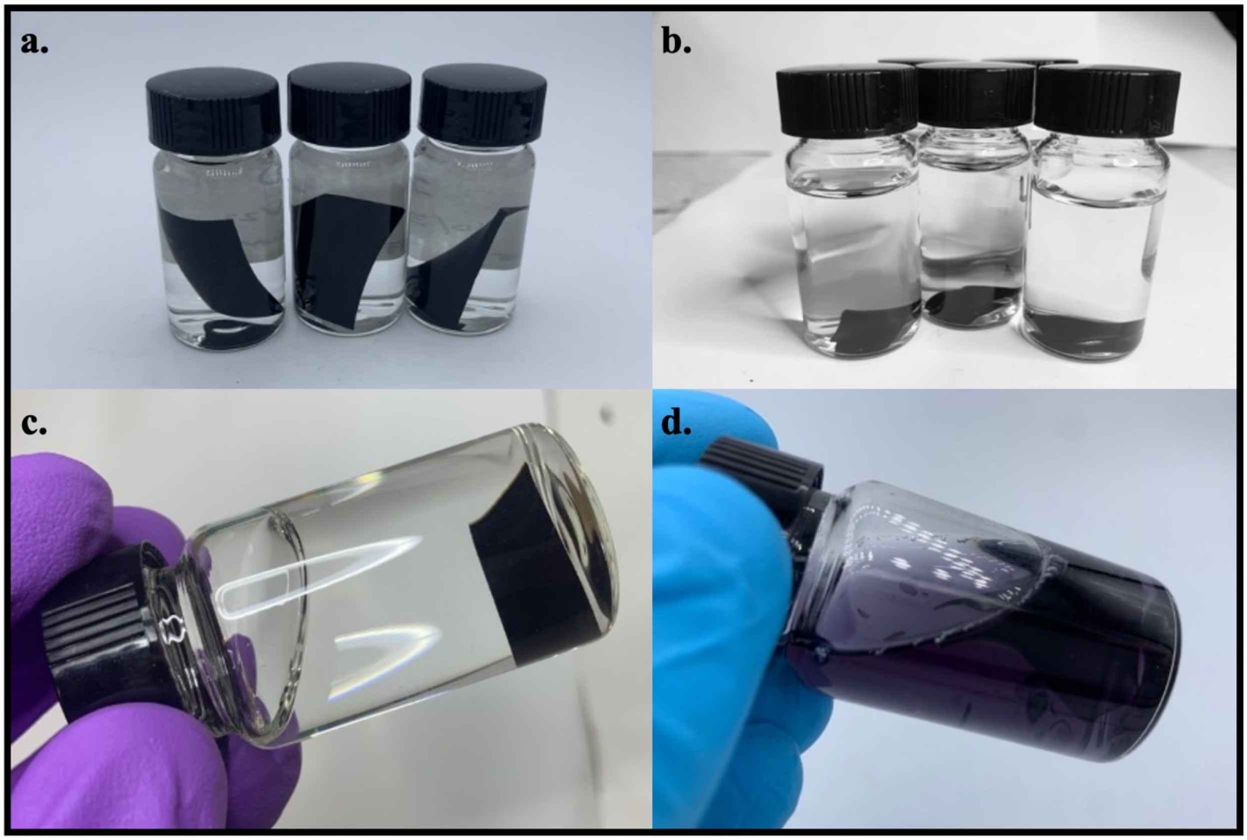

Figure S2: Macroscopic degradation of gelapin bioplastics. Gelapin coupons were incubated in PBS and SGF. Photographs of samples (a) before and (b) after 90 days of immersion in a 37 °C PBS solution (pH ~6.8). Photographs of samples (c) before and (d) after 24 days of heated agitation in simulated gastric fluid (SGF) .

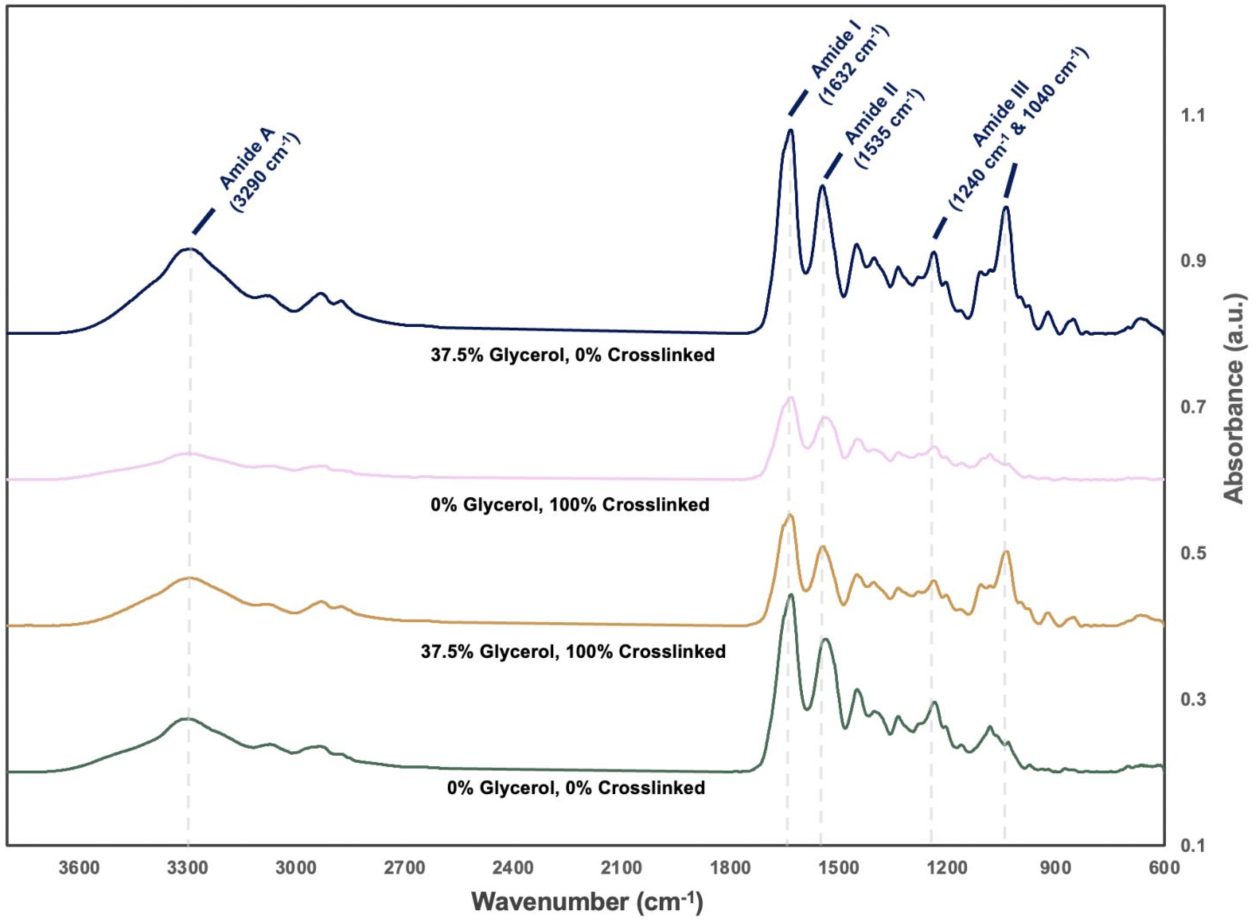

Figure S3: FTIR absorption analysis shows a broadening and reduction in Amide A peak intensity in the plasticized, uncrosslinked sample, which can indicate decreased intermolecular hydrogen bonds between gelatin strands. Additionally, the presence of glycerol suggests significant enhancement in the Amide III region (1030 cm^{-1} peak). This region tends to reflect C–N stretching vibrations coupled to N–H bend and may correlate to an increase in triple helix formation.

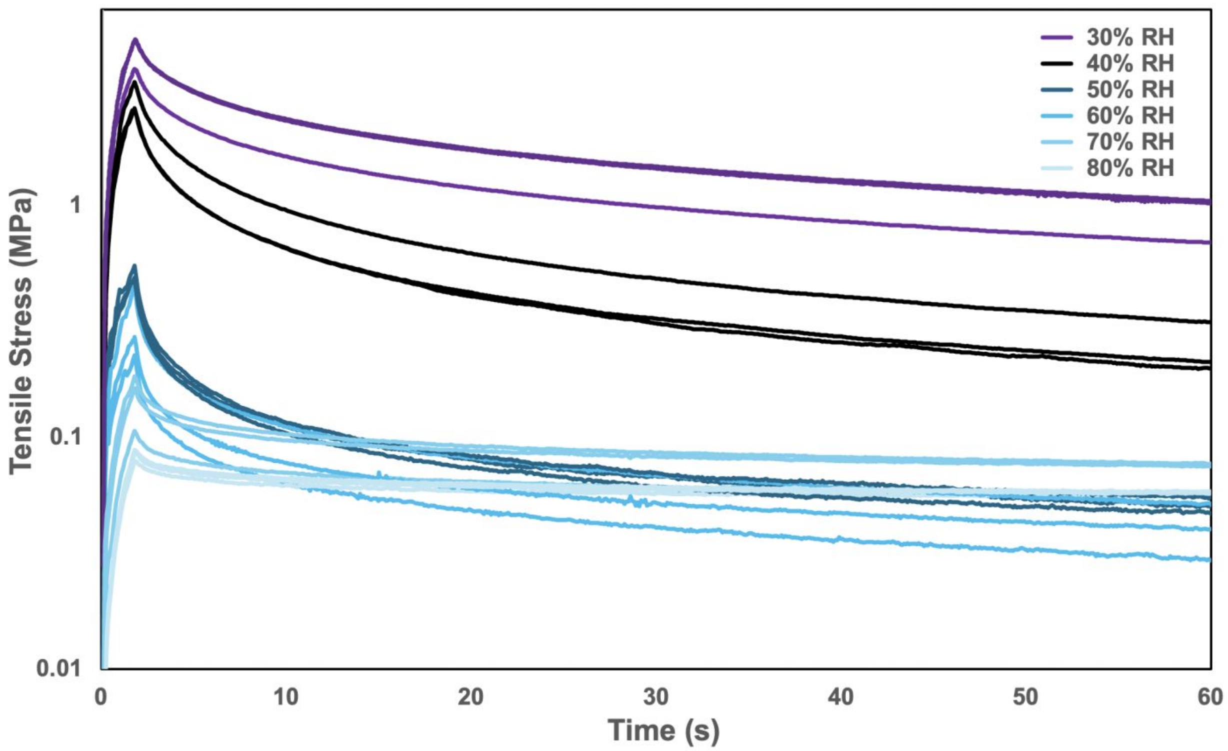

Figure S4: The stress relaxation plots of 100 mol% crosslinked, 37.5 wt% glycerol samples are included above. As expected, samples equilibrated at lower moisture levels reach a higher maximum stress during the constant strain period and demonstrate a slower stress decay once strain is held constant. Comparatively, as the diffused moisture content within samples rises, we see a more viscoelastic response: little stress is required to deform the sample, which then readily relaxes into a near-fixed deformation state once tensile forces are held constant. The differences in response profile between 30% RH and 80% RH samples reflects a highly sensitive, hygroscopic film.

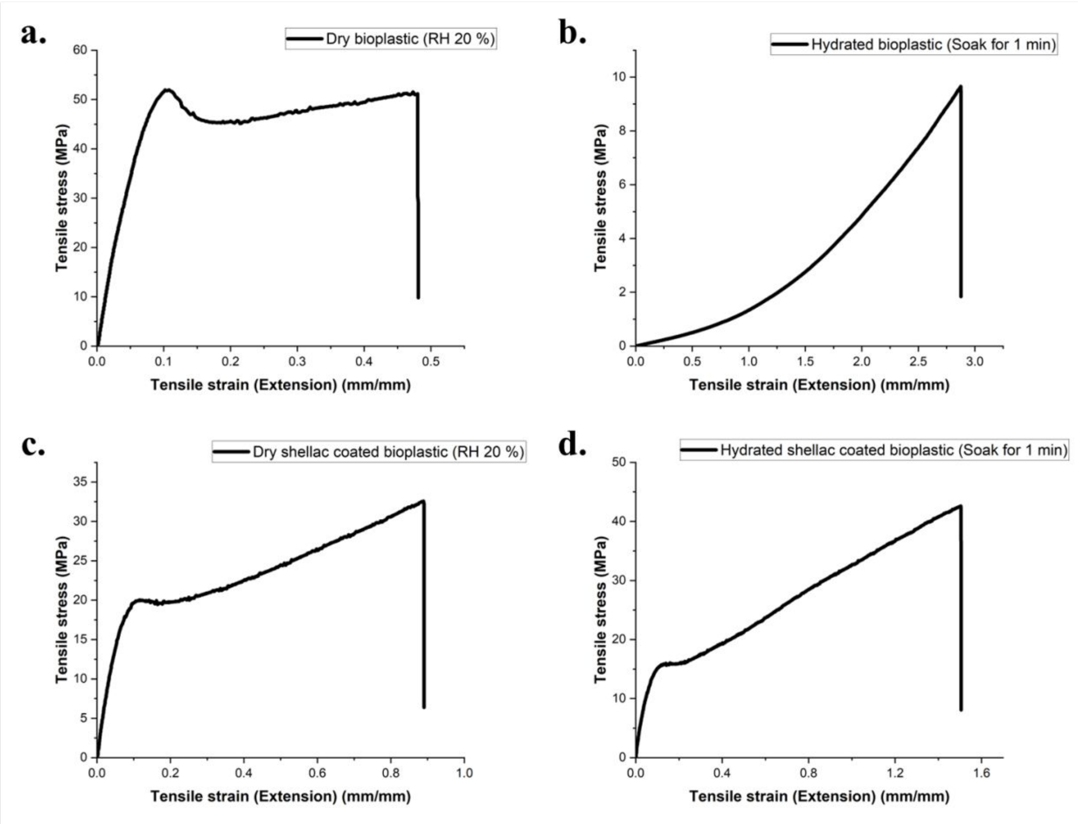

Figure S5: Stress-strain plots demonstrate the effect of spray coating samples of bioplastic (37.5% glycerol by wt. and 100% crosslinked) in a shellac coating. Sample types tested include (a) dry uncoated material, (b) hydrated uncoated material, (c) dry coated material, (d) and hydrated coated material.

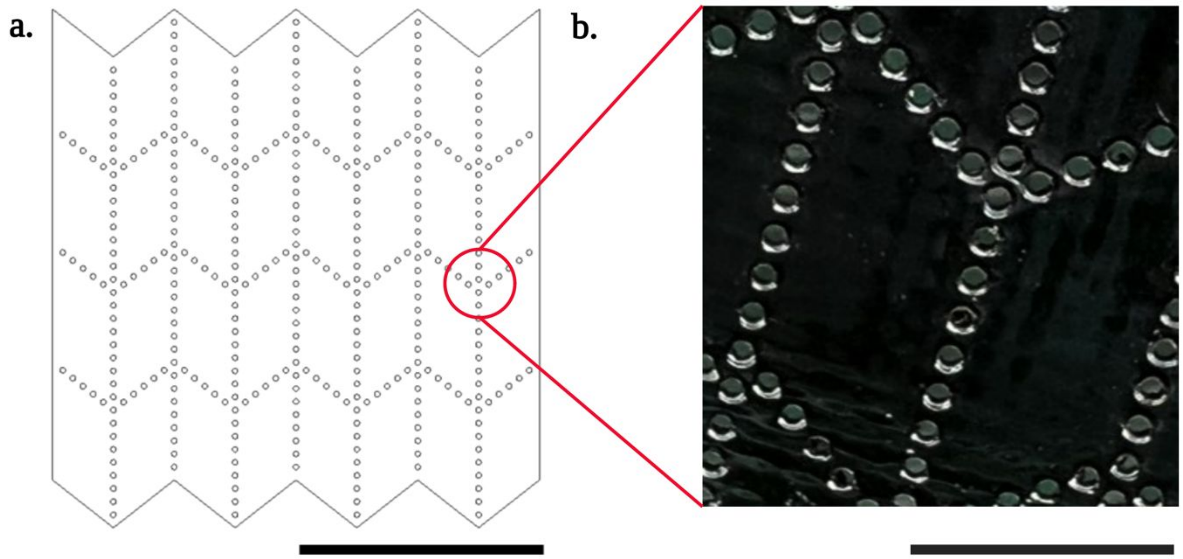

Figure S6: Perforation patterns for origami folding were laser cut with a LPKF ProtoLaser U4 UV laser (Tualatin, OR) operating at a wavelength of 355 nm, 80 kHz pulse frequency, 200 mm/s travel speed and 2 watts of power. Folding through-holes were designed with a diameter of 300 μm and a center-to-center distance of 1500 μm. (a) Sketch of a Miura-Ori cell array design with a 1 cm scale bar. (b) Magnified image of a laser-cut sample with a 2.5 mm scale bar, taken using an optical microscope.
